# Remarks on the Yellow Fever

**Published:** 1841-05-01

**Authors:** A. W. Upshur


### MEDICAL EXAMINER.

DEVOTED TO MEDICINE, SURGERY, AND THE COLLATERAL SCIENCES.

No. 18.] PHILADELPHIA, SATURDAY, MAY 1, 1841. [Vol. IV.

### ORIGINAL COMMUNICATION.

*Remarks on the Yellow Fever.* By A. W. Upshur, M. D.

To the Editors of the Medical Examiner.

It is still, I believe, a mooted point among medical men, whether yellow fever is or is not contagious. I have gleaned the opinions of several authors and other eminent men in the profession, upon this subject; which, with a few remarks, I submit to your inspection, and, if you deem them worthy, may insert them in your valuable periodical.

It will perhaps be proper, before proceeding to discuss the question of contagion, to give a brief outline of the nature and character of the disease under consideration.

This fever is known by many appellations. By Sauvages it has been called the *Typhus Icterodes*; by Cullen the *Typhus cum flavedine cutis*; among the French by the name of *Maladie de Siam*; and among the Spaniards by that of *Vomito Prieto* or *Black Vomit*. It is generally known in this country by the name of Yellow Fever.

The progress and violence of the yellow fever differ greatly, according to the force of its cause, the vigor and excitability of the patient, and the season of the yearr When it prevails epidemically in hot countries, and attacks young and robust individuals, lately arrived from temperate regions, the disorder commonly appears in its most aggravated form. In this, the patient first complains of lassitude, restlessness, slight sensations of cold and nausea; which symptoms are soon succeeded by strong arterial action, intense heat, flushing of the face, redness of the eyes, great pain and throbbing in the head, uneasiness and pain in the epigastric region, a white fur on the tongue, and a dry parched skin, with a quick, full, tense, and generally strong pulse, though it is sometimes oppressed and irregular.

These symptoms are speedily accompanied by frequent efforts to vomit, especially after swallowing food or drink, with discharges first of such matter as the stomach happens to contain, and afterwards of considerable quantities of bile, appearing first yellow and then green, sometimes tinged with blood; but in the progress of the disorder, with matter of darker colours; an increase of pain, heat, and soreness at the precordia also occurs, with constant wakefulness, and frequently with delirium more or less violent. This paroxysmal exacerbation, which has been called the inflammatory or febrile stage, generally lasts about thirty-six hours, but is sometimes protracted beyond this period, probably in consequence of either general or local inflammation. A remission then occurs, in which many of the symptoms subside, so as often to induce the belief that the fever is at an end, and recovery about to take place.

Frequently, however, the foundation of irreparable injury to the brain or stomach, has already been laid in the former paroxysm, and in such cases the remission is short and imperfect. During the remission, the pulse often returns almost to its natural state, the skin feels cool and moist, and the intellect, if previously disturbed, sometimes becomes clear; frequently, however, the patient remains in a quiet and stupid state, a symptom generally denoting great danger. After a certain interval, this remission is succeeded by another paroxysm, in which the symptoms of the first are renewed with great violence, accompanied with decided manifestations of cerebral disease, and also inflammation of some one of the abdominal viscera; the headach becomes intense, the delirium incontrollable, the eyes blood-shot and fiery, the stomach distressed with severe pain and ceaseless efforts to vomit, which, in many cases, are followed by hiccough, and repeated discharges of matter resembling turgid coffee, and also evacuations of a similar sort of matter from the bowels. Hence it is observed, that when these symptoms occur, indicating a violent affection of the stomach and bowels, the patient is, in general, sufficiently in possession of his intellect to know those about him, although excessive weakness often renders him incapable of mental exertion, and his inability even to raise his head, may induce the appearance of coma. In these cases, however, in which the brain has suffered greater injury than the stomach, the retching and the black vomit just described do not so commonly occur, but instead of them, low muttering delirium or coma, with convulsions of the muscles of the face and other parts of the body. About this time, also, the tongue is covered with a dark brown fur; yellowness of the skin and petechiæ make their appearance; the urine, when passed, has a putrid smell and dark colour; the feces likewise become putrid and very offensive; hæmorrhages sometimes takes place from the nostrils, gums, and various other internal surfaces; there is, in some cases, a suppression of urine, in others an involuntary discharge of it, and of the feces; portions of the skin assume a livid colour; the extremities grow cold, and life is gradually extinguished. This is a general outline of the yellow fever, when it appears in its most violent form; and in this form it sometimes proceeds with so much rapidity as to destroy the patient on the third or fourth day, or even sooner.

But the disorder frequently appears in a milder form at first, the course being protracted into several paroxysms, shorter at first, and followed by more distinct remissions; but afterwards increasing in violence and duration, when the disease terminates fatally. In these cases, death usually happens between the seventh and fifteenth days,

When the bodies of patients, in whom the affection of the head formed the principal feature of the disorder, have been inspected after death, the integuments of the brain have been found more or less inflamed, especially near the temporal bones; the vessels of the dura mater and of the pia mater are frequently found tinged with blood, which has also been sometimes extravasated into the brain. Effusions of a watery fluid have also occasionally been seen over the surface of the brain or in the ventricles. The volume of the brain is often increased, and the substance of it is, in some instances, more firm than usual; when cut, the vessels distributed through it have been so distended with blood, that the medullary part has become thickly spotted into red points, owing to oozing of blood from the divided vessels; and those vessels have occasionally been found ruptured, and that blood had consequently escaped into the brain.

Such is the disorganization which careful dissections have uniformly detected, in a greater or less degree, in those cases of yellow fever in which the predominant symptoms indicated a severe affection of the head. It accounts sufficiently for the occurrence of these symptoms and for the fatality which so often attends them, as well as for the derangement of mind, the loss of memory, and the impaired state of the sight, which are the frequent consequences of this disorder in those who escape with life, and from which they sometimes recover very slowly.

When the bodies of those who have died with the symptoms indicating inflammation of the stomach, have been dissected, the stomach has, in every instance, exhibited very evident signs of inflammation. In some instances the small intestines are found to have suffered from the same affection; the liver, and sometimes the kidneys, betray marks of great and destructive inflammation.

*Cause.*—The medical profession has for a long time been divided as to the origin and nature of yellow fever, some considering it to be a specific disease, and as originating in contagion; whilst others look upon it only as a form of the continued or autumnal fever of our country, and of course as originating from the same cause. We will first examine the grounds of its contagiousness, and then adduce some proof in favour of its *miasmatic* origin.

Contagion is a matter produced by a disease, capable, on application to a healthy body, of producing in it the same disease, and of causing it to produce contagious matter of the same kind. It is either fixed or volatile. The former produces its effect by contact only with the sick, or by inoculation, such as the vaccine virus, the syphilitic, &c.; the latter produces its effect in a manner unknown, but without contact with the sick, or with any thing *visibly* proceeding from them. When a person in good health is placed near one affected with morbid symptoms, and becomes affected in the same manner, the question arises, how was the disease produced in the second?

If, with all our care, we can discover no cause; if we find that in all the varying combinations of circumstances that have occurred for ages back, the morbid symptoms in question never appeared without the presence of a person previously affected in the same way; and that they are, in every change of circumstances, the invariable consequence of being present with a person so affected, the disease appearing wherever the sick go, and never where they do not; we are led to the conclusion, that the circumstances which produce the cause exist in the affected person, and we are induced to suppose that the person in health is influenced by something invisible passing from the sick, and this invisible influence we call contagion.

The belief in the existence of volatile contagion is, therefore, an *hypothesis*, resting for a support on its fitness to explain the spreading of a disease, and our inability to discover any other cause. That it is no more, will appear if we consider that we have no evidence whatever of the actual existence of volatile contagion. We *know* nothing of it; we only infer its existence from the single circumstance of the *spreading* of the disease.

If, however, we find that the disease is not the invariable consequence of being in the presence of a sick person; that it never follows unless this exposure be made in certain circumstances; that in these circumstances it arises whenever they occur, without the presence of the sick; we are entirely destitute of the only ground on which we can rest the hypothesis of contagion. Further, if in these circumstances we at length discover a cause capable of producing the symptoms observed, heretofore invisible, in consequence of being aëriform; if these symptoms uniformly appear in such circumstances, and in no other, the question is at an end; the cause of the first case is manifestly the cause of all that follow.

If a disease be propagated by a volatile contagion, the attendants must take it, for if they do not, we are destitute of the only ground upon which the hypothesis of contagion rests. They *must* communicate it to their attendants; these to others, and so on, until the disease becomes universal. The only thing which prevents this result, is a law of the system, impressed on it by the Creator, with all others necessary to the continuance of the race of man. This law is, that the system is so little disposed to be affected by volatile contagion, that, except very rarely, it produces no effect on a second application or exposure. By means of attendants exempt from its action, in consequence of having previously suffered, the communication between the sick and those in health who are still liable, is cut off, and the progress of the disease arrested. Without this restriction, it would not only be universal, but perpetual. It is, therefore, absolutely necessary to the very existence of the human race, that the volatile contagion, by which a mortal disease is propagated, be incapable, in general, of affecting the same person repeatedly.

However weak a volatile contagion may be, if it be capable of producing sickness, the attendants of the sick must be affected ; this, as we have before stated, being the only ground we have for believing it to exist. A disease, therefore, to which we are repeatedly liable, is not propagated by a volatile contagion. If a disease be propagated by a volatile contagion in *certain circumstances only*, it will spread until all in those circumstances are affected, unless it be stopped by insulating the sick by means of those who, having had it, are not again liable to be affected. We shall find all these characteristics of contagion in the small pox. First—we are not repeatedly liable to be affected by its contagion ; secondly—it would spread universally, unless stopped by insulating the sick, as above mentioned ; thirdly—in persons placed near the sick, the disease is almost invariably produced; fourthly—it is never produced under *any* circumstances (except by inoculation) without a near approach to persons affected with the disease; fifthly—it invariably spreads from the sick without regard to circumstances. We, therefore, believe this to be a contagious disease.

We will now apply these general remarks on contagion to yellow fever, and see whether it possesses the characteristics of a contagious disease. We are liable to repeated attacks of this disease. Dr. Rush, speaking of the yellow fever of 1793 in Philadelphia, says, "cases of re-infection were very common during the prevalence of this fever." In the year 1794, in which there was but little of the disease, he met with but few cases, the subjects of which had had the disease the year before.

Dr. Potter, in his memoir on contagion, states *that he has himself had this disease three times.* Mr. Doughty, a British surgeon, who served eight years in the West Indies, mentions the case of the 85th Regiment, which suffered dreadfully from the concentrated form of yellow fever in Spanish Town in 1805. The next season they escaped it entirely in Fort Augusta; but again, in 1807, they were nearly annihilated in Kingston. At the latter time and place, Mr. Doughty was himself at the brink of the grave, from an attack of the yellow fever, though seven years previously he asserts that he had it in its concentrated form in the same town. Dr. Dickerson, in a communication to Dr. Johnson respecting the yellow fever of 1808 in Mariegalante, says, "many of the old as well as the new troops were seized with the fatal fever; indeed, the *worst cases were second attacks* of this disease ;" and, therefore, it is not propagated by volatile contagion.

Numberless facts support this conclusion:—first, great numbers who are near the sick, attending on them, escape the disease entirely ; secondly, it often is, and may at any time be produced by certain circumstances, without the presence of persons previously affected ; thirdly, when the sick are carried out of these circumstances, or removed beyond the influence of the cause there generated, no person who approaches them will become affected.

Great numbers who are near the sick, attending on them, escape entirely. A multitude of instances of this might be stated. The following are from Dr. Johnson's account of the yellow fever of 1820 in Philadelphia. The family of Hays (in which occurred the first case reported to the board of health) occupied a single room. It consisted of himself, wife, and three children, all of whom *shared the same bed during his illness.* The other tenants of the house, the neighbours, and his acquaintances frequently visited him while sick, and a number of persons assembled at the house to attend his funeral ; *not an individual thus exposed, sickened.* A rigger contracted the disease, sickened and died. Two families occupied the house, the individuals of which were obliged to pass through where this man lay sick, in going in and out of the house. The neighbours, also, were frequently with him. No one took the disease from him, though the alley was, at that time, very filthy and offensive. Other instances of the same kind are mentioned by Dr. Johnson.

Dr. Ferguson, in his essay on marsh poison, states, that it *often* happened that the soldiers belonging to the barracks at Monk's Hill, Antigua, who in perfect health mounted guard in the night, among the marshes at the foot of the hill, were seized while standing sentry, and when carried back to the barracks, expired with the black vomit, in thirty hours from the commencement of the attack. And, notwithstanding this, not a single case of yellow fever, nor of fever of any kind, occurred among the inhabitants of Monk's Hill, who were not obliged to sleep out of the garrison, or to take the duties in the marshes below. This disease, then, often is, and may at any time be produced by certain circumstances, without the presence of persons previously affected.

[We shall have some comments to offer on this paper, when concluded.]—Eds.

